# Supplementary material for: Distinct multiple fermionic states in a single topological metal
Source: Nat Commun. 2018 Aug 1;9:3002. doi: 10.1038/s41467-018-05233-1 (PMC6070493; doi:10.1038/s41467-018-05233-1)
Supplement: Supplementary file 1 — Supplementary Information [file 41467_2018_5233_MOESM1_ESM.pdf]

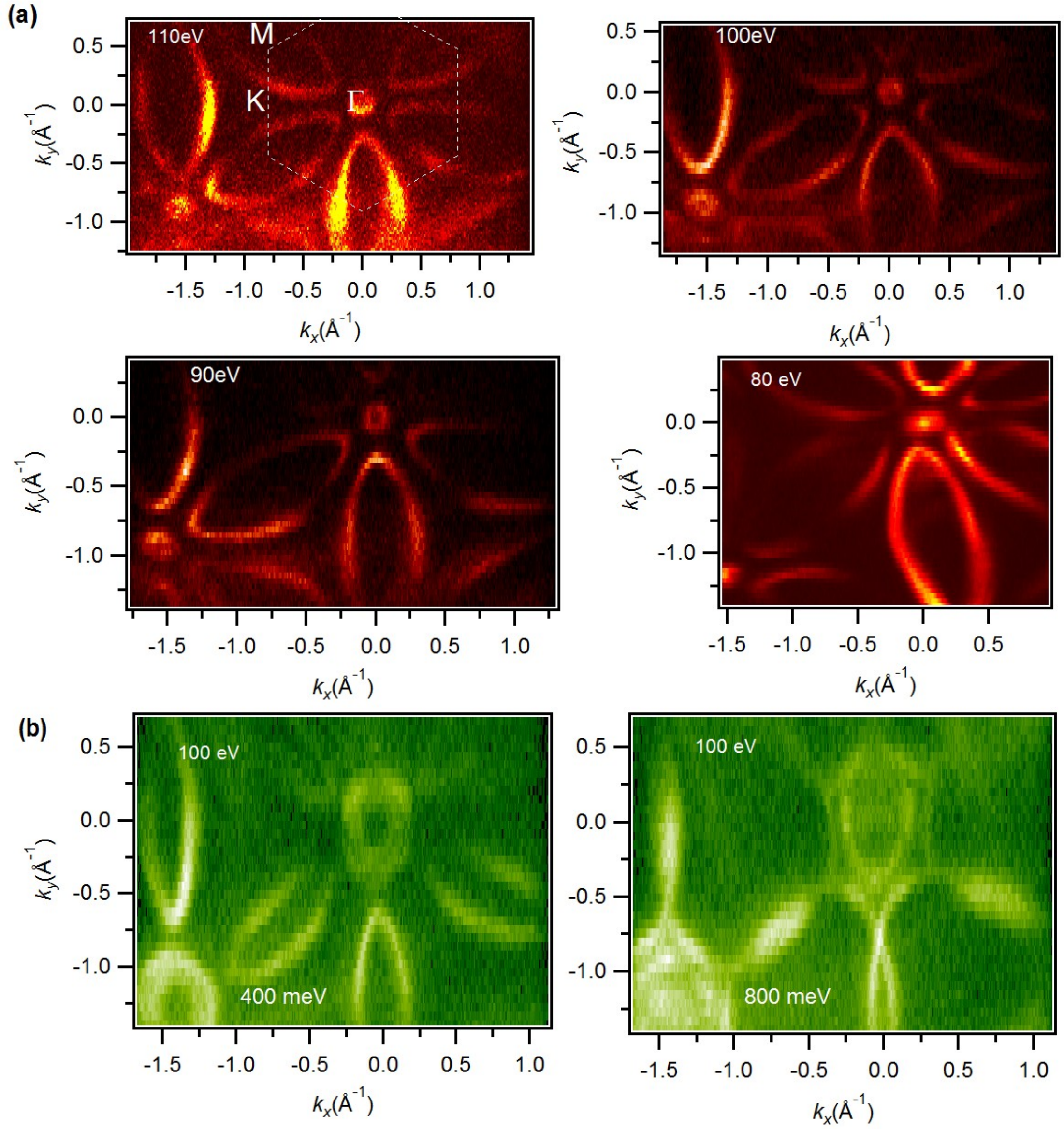


**Supplementary figure** 1: Fermi surface maps of $Hf_2Te_2P$. (a) Fermi surface maps over a wide incident photon energy window (80 - 110 eV) with 10 eV energy steps. The hexagonal Fermi surface is clearly observed; the white dashed hexagon in the 110 eV plot serves as a guide to the eyes. High-symmetry points are also noted in the 110 eV plot. (b) Constant energy contour plots at different binding energies.

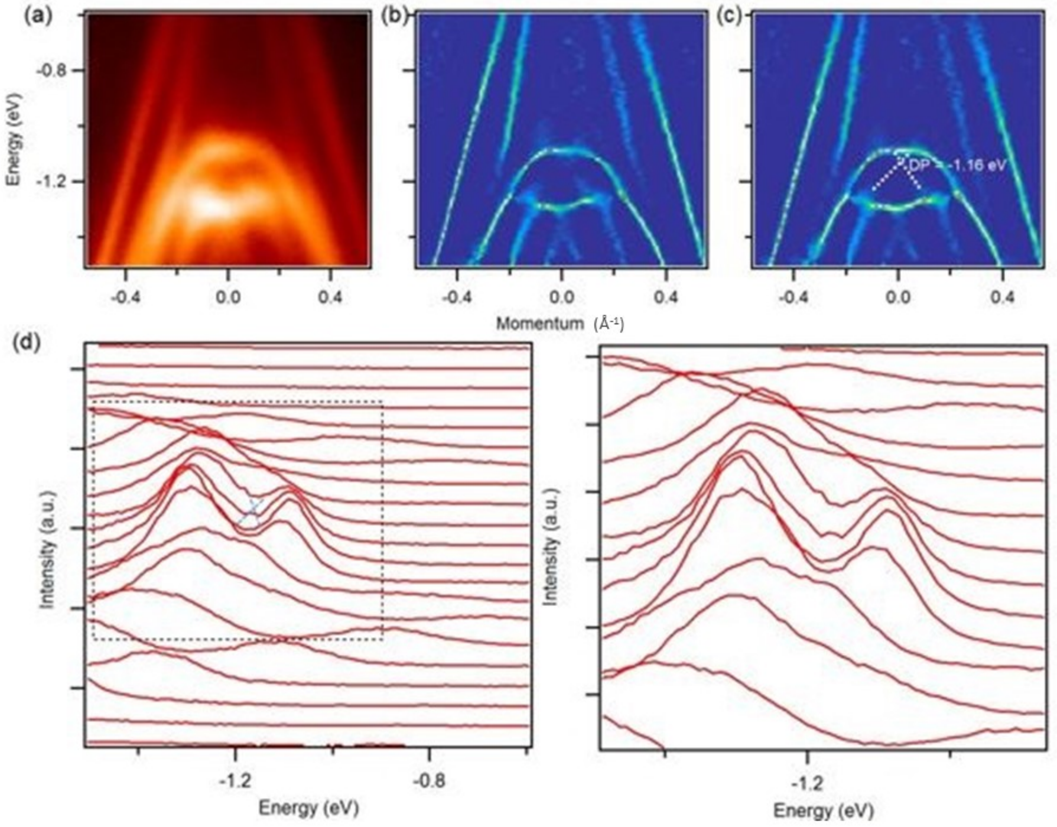


**Supplementary figure** 2: Observation of additional Dirac crossing. (a) Measured dispersion map along the K-Γ-K direction (zoomed view near the Dirac point) using a photon energy of 90 eV. (b) Second derivative plot of the same figure using curvature method [1]. (c) Similar plot as 2(b) with a white dashed lines as guides for the eyes to highlight the Dirac point at about 1.1 eV below the Fermi level. (d) Energy distribution curves of the dispersion map shown in (a). The dashed lines serve as guides for the eyes. Zoomed view of the rectangular box near the Dirac point (right).

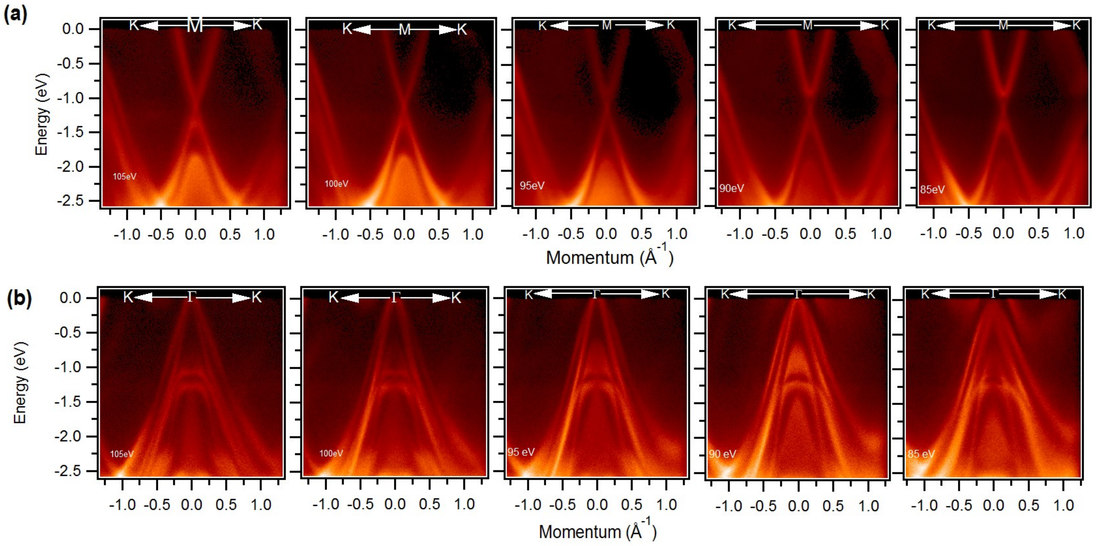


**Supplementary figure** 3: Photon energy dependent dispersion maps. (a) ARPES measured dispersion maps along the K-M-K direction using photon energies of 85 -105 eV with 5 eV energy steps. (b) Dispersion maps along the K-Γ-K direction using various photon energies as indicated in the plots.

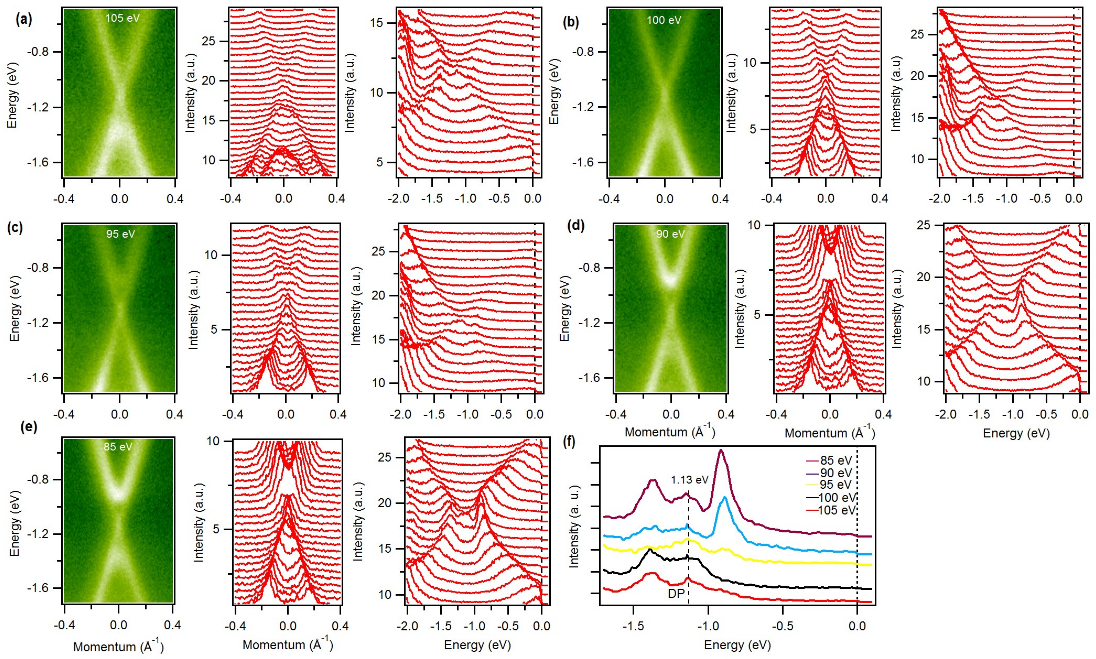


**Supplementary figure** 4: Momentum and energy distribution curves along the K-M-K direction. (a)-(e) Dispersion maps near the Dirac point along the K-M-K direction and their corresponding momentum distribution curves (MDC) and energy distribution curves (EDCs). Here, we unambiguously observe the Dirac point at the high symmetry point M. (f) Combined EDCs at the M point. The Dirac point does not change with photon energy.

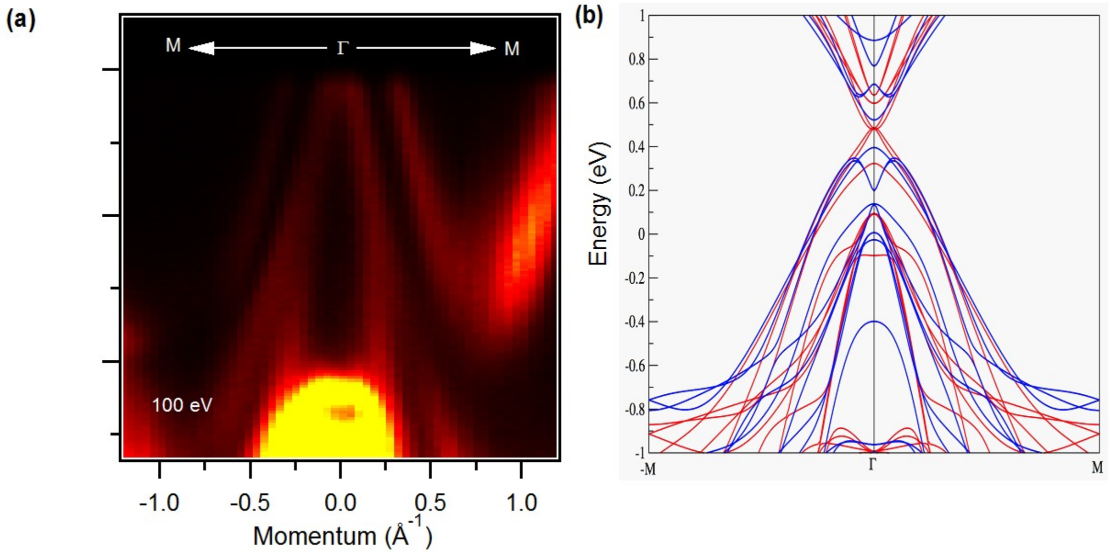


**Supplementary figure** 5: Observation of topological Dirac cone. (a) ARPES measured dispersion map along the M-Γ-M direction using a photon energy of 100 eV. (b) Calculated bands along the M-Γ-M directions using the bulk conventional unit cell.

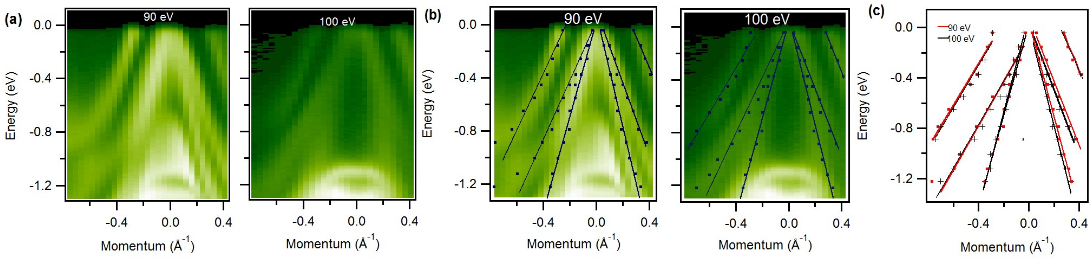


**Supplementary figure** 6: Observation of surface state. (a),(b) 90 and 100 eV photon energy dependent dispersion maps and MDC fits plots overlaid along the M-Γ-M direction, respectively (see also main text Figure 2(c)). (c) Combined MDC fits of the 90 and 100 eV plots. The red dots and black crosses correspond to the 90 and 100 eV dispersion maps, respectively.

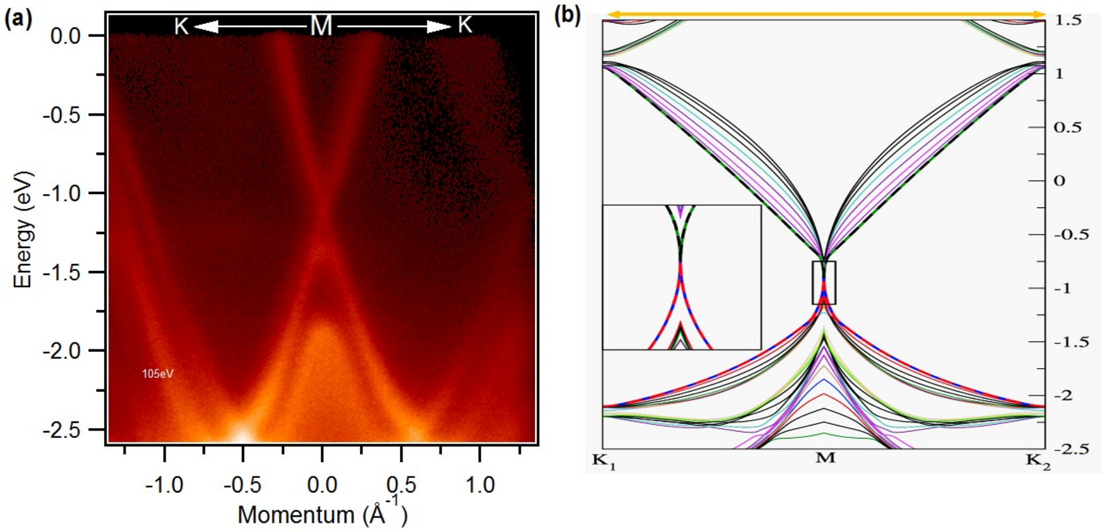


**Supplementary figure** 7: Linearly dispersive bands within a wide energy window. (a) ARPES measured dispersion map along the K-M-K direction using a photon energy of 105 eV. (b) Calculated bands along the K-M-K direction. Linearly dispersive states are clearly observed in a wide energy window. A Dirac crossing of the surface states at the M point is clearly visible (see inset).

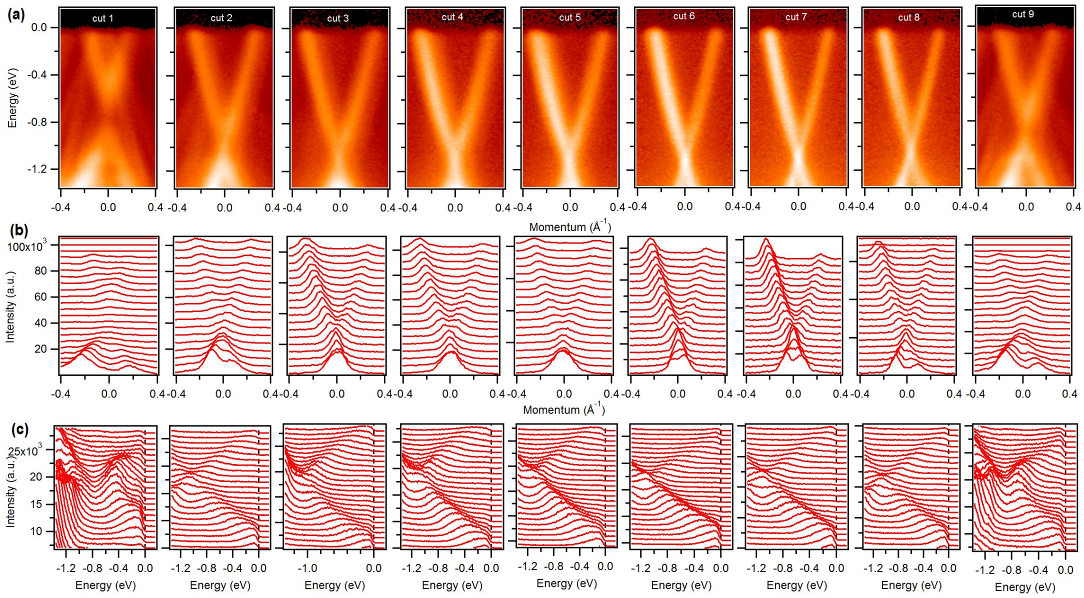


**Supplementary figure** 8: Evolution of the Dirac crossing along the Dirac-node arc. (a)-(c) Measured dispersion maps, MDCs, and EDCs plots along the Dirac-node arc, respectively.

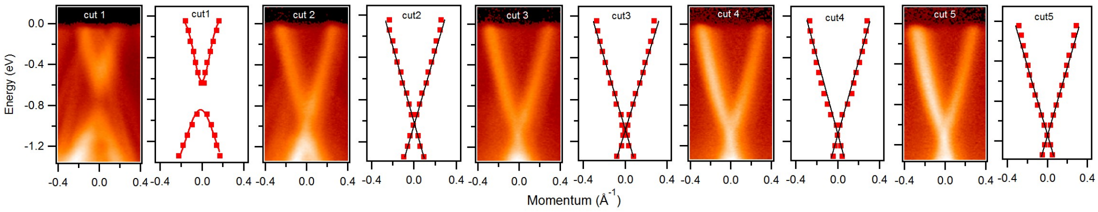


**Supplementary figure** 9: MDC fits along the Dirac-node arc. Dispersion maps and MDC fits of cuts 1, 2, 3, 4 and 5 are placed side by side to observe the upward movement of the Dirac point along the Dirac arc.

**Supplementary note 1: Fermi surface and observation of weak topological state:**
Supplementary Figures 1(a),(b) show the Fermi surface maps obtained with different photon energies and the constant energy contour plots, respectively. At higher binding energies, one can clearly identify the two concentric hexagons around the Γ-point of the Brillouin zone (BZ). The two hexagons correspond to the two hole-like bands crossing the Fermi level. This indicates the presence of multiple topologically protected bands around the Γ-point of the BZ and, as a consequence, we find a pair of linearly dispersive states above the Fermi level (see main text Fig. 4(b)). Furthermore, our calculations suggest the presence of a distinct Dirac-like state at the same momentum position (at the zone center (Γ-point)) but at a different energy at about 1.1 eV below the Fermi level. However, it is not well discernible due to the broad ARPES intensity at this momentum position (see supplementary Fig. 2(a)). Therefore, we have created a second derivative plot of supplementary Fig. 2(a) using the curvature method (see supplementary Fig. 2(b),(c)) [1]. Here, we can observe the distinct second Dirac cone. We present energy distribution curves (EDCs) near the Dirac cone in supplementary Fig. 2(d), in order to confirm our observation. The exact location of the Dirac point is around 1.16 eV below the Fermi level (see SF 2(c),(d)). Furthermore, the lower part has a Λ-type dispersion where the upper part resembles an $m$-shaped dispersion which is distinct from a trivial insulator and in agreement with the data reported by various groups [2, 3].

**Supplementary note 2: Experimental observation of surface states.**
The photon energy dependent ARPES dispersion maps allow us to identify the band origin unambiguously. Therefore, we present dispersion maps along the K-M-K and K-Γ-K directions (see supplementary Figure 3(a),(b)) over a wide energy range (85 - 105 eV) with energy step of 5 eV. Within this wide energy window the linearly dispersive bands around the M point and the two hole-like bands around the Γ-point do not show any dispersion in supplementary Figure 3(a),(b), respectively. The Dirac state is further confirmed by the MDC and EDC plots shown in supplementary Fig. 4. Therefore, we can conclude that these bands are surface originated.

**Supplementary note 3: Linearly dispersive bands over a wide energy window.**
Supplementary Figure 5(a) represents a dispersion map along the M-Γ-M direction. As ARPES can only probe occupied bands, it is technically not feasible to observe the Dirac cone state at the Γ-point which is located above the Fermi level. To confirm the presence of the Dirac state at this high symmetry point, we present band calculations along the M-Γ-M direction (see supplementary Fig. 5(b)). The band inversion takes place around 0.5 eV above the Fermi level and we can clearly observe its surface origin (shown in red color). MDC fit curves can be useful to observe surface originated bands more quantitatively. Supplementary Fig. 6(a),(b) presents the measured dispersion maps along the M-Γ-M direction and overlaid with MDC fits, respectively. Supplementary Fig. 6(c) shows the MDC fit plots of 90 and 100 eV dispersion maps along the Γ-M direction. The fitted MDC peak approximately shows the linear dispersion within this momentum window. Therefore, based on our calculations and experimental observations we can attribute these bands to be surface originated. Supplementary Figures 7(a),(b) show the measured and calculated band dispersions along the K-M-K direction, respectively. Here, we observe the linearly

dispersive bands over an energy window more than 2.3 eV wide which is larger than that of any other known topological material. The red color in supplementary Fig. 7(b) represents the surface states.

**Supplementary note 4: Evolution of the Dirac crossing along the Dirac node arc.**
The Dirac points along the Γ-M-Γ direction form a nodal-arc in energy-momentum space. The center of the arc possesses a sharp Dirac crossing (see Fig. 3c). While moving towards the zone center from either side of the M point, the Dirac point moves upward with energy and eventually gaps out as shown by the MDCs and EDCs presented in supplementary Fig. 8 and MDC fits of cuts 1, 2, 3, 4 and 5 in supplementary Fig. 9.

# Supplementary References

[1] Zhang, P., Richard, P., Qian, T., Xu, Y.-M., Dai, X. & Ding, H. A. precise method for visualizing dispersive features in image plots. Rev. Sci. Instrum. **82**, 043712 (2011).

[2] Neupane, M. *et al.* Observation of Topological Nodal Fermion Semimetal Phase in ZrSiS. Phys. Rev. B **93**, 201104, (2016).

[3] Chen, C. *et al.* Dirac line nodes and effect of spin-orbit coupling in the non-symmorphic critical semimetals MSiS (M = Hf,Zr). Phys. Rev. B **95**, 125126 (2017).
